# Structural insights into peptide self‐assembly using photo‐induced crosslinking experiments and discontinuous molecular dynamics

**DOI:** 10.1002/aic.17101

**Published:** 2020-11-07

**Authors:** Samuel J. Bunce, Yiming Wang, Sheena E. Radford, Andrew J. Wilson, Carol K. Hall

**Affiliations:** ^1^ School of Chemistry University of Leeds Leeds UK; ^2^ Astbury Centre for Structural Molecular Biology University of Leeds Leeds UK; ^3^ Department of Chemical and Biomolecular Engineering North Carolina State University Raleigh North Carolina USA; ^4^ Department of Chemical and Biological Engineering Princeton University Princeton New Jersey USA; ^5^ School of Molecular and Cellular Biology University of Leeds Leeds UK

**Keywords:** amyloid‐forming peptide, discontinuous molecular dynamics, peptide self assembly, photo‐induced crosslinking

## Abstract

Determining the structure of the (oligomeric) intermediates that form during the self‐assembly of amyloidogenic peptides is challenging because of their heterogeneous and dynamic nature. Thus, there is need for methodology to analyze the underlying molecular structure of these transient species. In this work, a combination of fluorescence quenching, photo‐induced crosslinking (PIC) and molecular dynamics simulation was used to study the assembly of a synthetic amyloid‐forming peptide, Aβ_16‐22_. A PIC amino acid containing a trifluormethyldiazirine (TFMD) group—Fmoc(TFMD)Phe—was incorporated into the sequence (Aβ*_16–22_). Electrospray ionization ion‐mobility spectrometry mass‐spectrometry (ESI‐IMS‐MS) analysis of the PIC products confirmed that Aβ*_16–22_ forms assemblies with the monomers arranged as anti‐parallel, in‐register β‐strands at all time points during the aggregation assay. The assembly process was also monitored separately using fluorescence quenching to profile the fibril assembly reaction. The molecular picture resulting from discontinuous molecule dynamics simulations showed that Aβ_16‐22_ assembles through a single‐step nucleation into a β‐sheet fibril in agreement with these experimental observations. This study provides detailed structural insights into the Aβ_16‐22_ self‐assembly processes, paving the way to explore the self‐assembly mechanism of larger, more complex peptides, including those whose aggregation is responsible for human disease.

## 1 INTRODUCTION

Self‐assembling peptides can be difficult to study in vitro because of their hydrophobicity and propensity to aggregate into high order assemblies.^1^, ^2^, ^3^, ^4^ Consequently, shorter synthetic peptide fragments from longer amyloidogenic sequences offer convenient model systems with which to explore peptide self‐assembly.^5^, ^6^ A case in point is the Gly‐Asn‐Asn‐Gln‐Gln‐Asn‐Tyr (GNNQQNY) sequence, taken from the prion‐determining domain (PrD) of the 635 residue Sup35 yeast protein.^7^, ^8^ Eisenberg and co‐workers established in 2001 that in aqueous conditions this sequence self‐assembles into highly ordered fibril structures that display the characteristic cross‐β X‐ray diffraction pattern of amyloid.^6^ The short nature of GNNQQNY enabled the formation of micro‐crystals suitable for electron diffraction, which demonstrated that in the crystals the peptide had formed a parallel in‐register β‐sheet structure involving a steric zipper with a dry interface between the interdigitated side‐chains.^7^ GNNQQNY clearly demonstrates that peptide fragments can be useful models of longer amyloid sequences, revealing atomic level information about short segments that may be relevant to the assembly of their longer peptide counterparts.

One of the most widely studied short amyloidogenic sequences is Aβ_16‐22_ (Ac‐Lys‐Leu‐Val‐Phe‐Phe‐Ala‐Glu‐CONH_2_), a seven‐residue peptide that comes from the central fibril‐forming region of the Aβ sequence associated with Alzheimer's disease.^5^ Aβ_16‐22_ is one of the smallest Aβ fragments that forms fibrils with a cross‐β structure and can be readily synthesized using solid phase peptide synthesis (SPPS). Substitutions within this region of full‐length Aβ peptide have been shown to affect its aggregation propensity in vitro and in vivo, highlighting the importance of the motif.^9^, ^10^, ^11^ These findings make Aβ_16‐22_ a convenient model with which to explore the underlying steps in an aggregation mechanism. The noted ability of Aβ_16‐22_ to form a range of supramolecular structures at different pH values further highlights the value of using Aβ_16‐22_ to understand the fundamental molecular mechanisms of peptide self‐assembly and the molecular origins of fibril polymorphism.^12^, ^13^ Tycko and co‐workers used solid‐state NMR, transmission electron microscopy (TEM) and X‐ray diffraction to establish that at neutral pH Aβ_16‐22_ formed in‐register, anti‐parallel fibril structures that displayed green birefringence when bound to Congo‐Red.^5^ In X‐ray diffraction experiments the fibrils displayed periodic reflections at 4.9 and 9.9 Å, characteristic of the spacing between β‐strands and β‐sheet layers, respectively.^14^, ^15^

A key goal in studying Aβ_16‐22_ is to understand the transitions that occur during self‐assembly. Petty and Decatur used isotope‐edited IR to establish that at neutral pH, Aβ_16‐22_ assembles with monomers adopting an initial β‐strand alignment that is not identical to the final equilibrium alignment, that is, there is some β‐sheet reorganization during the self‐assembly process.^16^ Lynn and co‐workers demonstrated that at both acidic and neutral pH, Aβ_16‐22_ passes through an intermediary out‐of‐register ribbon‐like structure.^13^ The time taken to reach the final fibril alignment (measured by CD) was different at each pH: at neutral pH Aβ_16‐22_ reached a plateau after 5 days, whereas at acidic pH a lag phase of 4 days was observed, with the final plateau being reached after 10 days.^13^ Aβ_16‐22_ has also been shown to form micrometer sized particles with a high concentration of peptides in a liquid‐like state.^17^, ^18^ These particles are metastable (containing around 20–33% β‐sheet content) and can undergo a phase transition to form nanotubes or fibrils at certain temperatures and pH values. In all cases, it should be noted that changes in temperature, peptide concentration and ionic strength can have a significant impact on the kinetics of Aβ_16‐22_ aggregation, confounding comparison between different studies.^12^, ^13^, ^18^, ^19^

Since the transient intermediates that form during the early stages of peptide self‐assembly can be difficult to characterize experimentally, molecular dynamics (MD) simulations can provide useful insights.^20^, ^21^, ^22^ The first studies, carried out by Klimov and Thirumalai, demonstrated that monomeric Aβ_16‐22_ preferentially adopts either a random coil or extended β‐sheet conformation.^20^ These structures then progress through an obligatory α‐helical intermediate prior to forming stable anti‐parallel, in‐register oligomers. Later studies also observed that Aβ_16‐22_ monomers adopt primarily random coil conformations, although the presence of an α‐helical intermediate was disputed.^21^, ^22^ These simulations also highlighted the complex pathways that Aβ_16‐22_ accesses during the self‐assembly process and that the anti‐parallel, in‐register structure is the preferred structure due to its stability. Hall and co‐workers^23^ applied discontinuous molecular dynamics (DMD) and a coarse‐grained protein model (PRIME20) to demonstrate that at high simulation temperatures, a system of 48 monomeric Aβ_16‐22_ peptides aggregates via classical nucleation and growth into a highly‐ordered structure with monomers organized as anti‐parallel, in‐register β‐strands in agreement with the solid‐state NMR measurements reported by Tycko and co‐workers.^5^ At lower simulation temperatures, Aβ_16‐22_ first forms β‐sheet‐rich oligomers which then merge and rearrange into a large fibril. Later, Hall and co‐workers^24^ combined the DMD/PRIME20 simulation with classical nucleation theory to construct a thermodynamic (solubility) phase diagram for Aβ_16‐22_, which agrees well with in vitro solubility measurements. Recently, PRIME20/DMD simulations were combined with electrospray ionization ion‐mobility spectrometry mass‐spectrometry (ESI‐IMS‐MS) measurements to reveal structural insights on the secondary nucleation mechanism of Aβ_1‐40_ peptide on Aβ_16‐22_ fibril surface.^25^

In this work, we combine experimental and molecular simulation to understand and characterize, at the molecular level, the transitions that Aβ_16‐22_ undergoes as it self‐assembles. We used photo‐induced crosslinking (PIC) combined with ESI‐IMS‐MS to derive insights on the noncovalent organization of Aβ_16‐22_ during different phases of its self‐assembly reaction. The simulation results are in close agreement with experimental results, and indicate that the dominant pathway by which Aβ_16‐22_ assembles involves monomers organized as anti‐parallel, in‐register β‐strands at all time points. The results highlight the power of combining PIC with ESI‐IMS‐MS/MS, fluorescence quenching and DMD simulations to study kinetic intermediates in peptide self‐assembly, and paves the way for exploring the self‐assembly of larger, more complex peptides, including those directly relevant to amyloid disease.

## 2 MATERIALS AND METHODS

### 2.1 Solid Phase Peptide Synthesis of Aβ_16‐22_

All amino acids, 4‐(trifluormethyldiazirine)phenylalanine (TFMD‐Phe), Aβ_16‐22_ (Ac‐Lys‐Leu‐Val‐Phe‐Phe‐Ala‐Glu‐NH_2_)_,_ Aβ_16–22_ N‐terminally labeled with tetramethylrhodamine (TAMRA) including a 6‐aminohexanoic acid linker (Ahx); TAMRA‐Ahx‐Aβ_16‐22_ and Aβ_16‐22_ functionalized TFMD‐Phe; Aβ*_16–22_ (Ac‐Lys‐Leu‐Val‐Phe‐(TFMD)Phe‐Ala‐Glu‐NH_2_) were prepared as described previously.^25^, ^26^, ^27^

### 2.2 Fluorescence quenching assays

Fluorescence quenching assays were carried out as described previously.^25^ Briefly Aβ_16–22_ was spiked with 5% (w/w) TAMRA‐Ahx‐Aβ_16–22_ (total peptide concentration, 20 or 40 μM) in 100 mM ammonium bicarbonate buffer (pH 7.4) with a final concentration of 2% (v/v) DMSO. Samples were placed in quartz cuvettes and analyzed using a temperature‐controlled fluorimeter at 37°C. Time points were taken every 30 s for the duration of the experiment. The TAMRA fluorophore was excited at 520 nm, and emission was recorded at 600 nm to reduce the inner filter effect.

### 2.3 TEM analyses

Samples were prepared and analyzed by TEM as described previously.^25^ Briefly, TEM images were taken at the stated time points and at the end of each experiment by removing 5 μl from the assembly reaction and incubating this sample on carbon–formvar grids for 30 s before staining with 2% (w/v) uranyl acetate solution for an additional 30 s. Images were taken on a JEM‐1400 (JEOL Ltd., Tokyo, Japan) or a Tecnai F12 TEM. (FEI, Hillsboro, OR) TEM. Images were taken using either an ATM charge‐coupled device (CCD) camera or a Gatan UltraScan 1,000 XP (994) CCD camera (JEM‐1400) or an UltraScan 100XP (994) CCD camera (Tecnai F12). Once taken, images were processed using ImageJ (National Institutes of Health [NIH]).

### 2.4 Photo‐induced cross‐linking

Aβ*_16–22_ (40 μM total peptide concentration) in 100 mM ammonium bicarbonate buffer (pH 7.4) with a final concentration of 1% (*v/v*) DMSO was incubated quiescently in Eppendorf tubes for 0 min, 5 min, or 24 hr. Samples were then irradiated for 30 s using a light‐emitting diode lamp at 365 nm,^28^ then removed, lyophilized overnight, redissolved in hexafluorisopropanol (HFIP) for at least 2 hr, and vortexed to ensure disaggregation. HFIP was then removed under a stream of N_2_, and the sample was re‐suspended in 50:50 (*v/v*) MeCN/H_2_O containing 0.05% (*v/v*) formic acid to a final peptide concentration of ~40 μM. Cross‐links were then analyzed using ESI‐IMS‐MS/MS as described below.

### 2.5 Electrospray ionization ion‐mobility spectrometry mass‐spectrometry (ESI‐IMS‐MS/MS)

All samples were prepared as described above and left to incubate at 37°C without agitation for 5 min. A SYNAPT HDMS quadrupole time‐of‐flight MS (Micromass UK Ltd., Waters Corp., Manchester, UK), equipped with a TriVersa NanoMate (Advion Biosciences, Ithaca, NY) automated nano‐ESI interface was used in this study. The instrument has a traveling‐wave IMS device situated in between the quadrupole and the time‐of‐flight analyzer. Samples were analyzed by positive ionization nano‐ESI, with a capillary voltage of 1.4 kV and a nitrogen‐nebulizing gas pressure of 0.8 psi. The following instrumental parameters were set: cone voltage, 60 V; source temperature, 60°C; backing pressure, 4.7 mbar; ramped traveling speed, 7 to 20 V; traveling wave speed, 400 m s^−1^; IMS nitrogen gas flow, 20 ml min^−1^; IMS cell pressure, 0.55 mbar. The mass/charge ratio (*m*/*z*) scale was calibrated using aq. CsI cluster ions. Collision Cross Section (CCS) measurements were estimated using a calibration obtained by analysis of denatured proteins (cytochrome c, ubiquitin, and alcohol dehydrogenase) and peptides (tryptic digests of alcohol dehydrogenase and cytochrome c), with known CCSs obtained elsewhere from drift tube ion mobility measurements.^29^, ^30^ The CCS (Ω) of the peptide monomers/oligomers was then calculated according to Equation (1):

(1) $$\Omega \left({\overset{˚}{A}}^{2}\right)=A\times {\left(t_{D}\right)}^{B}\times z\times \sqrt{\frac{1}{m_{\mathrm{ion}}}+\frac{1}{m_{\mathrm{gas}}}}$$

where A is the determined calibration constant, *z* is the charge state of the ion, *B* is the exponential factor (determined experimentally), *t*_D_ is the corrected absolute drift time, *m*_ion_ is the mass of the ion and *m*_gas_ is the mass of the gas used in the ion‐mobility cell (N_2_). Data were processed using MassLynx v4.1 and Driftscope software supplied with a mass spectrometer.

### 2.6 DMD simulation

In this work, three independent DMD/PRIME20 simulations were carried out for 12 μs in the canonical (NVT) ensemble. The two major nonbonded interactions of the PRIME20 model are the directional square‐well backbone hydrogen bonding interaction and the nondirectional square‐well potential interaction between two sidechain beads. The potential energy parameters between the 20 different amino acids include 210 independent square‐well widths and 19 independent square‐well depths derived by using a perceptron learning algorithm that optimizes the energy gap between 711 known native states from the Protein Data Bank and decoy structures.^31^ The Andersen thermostat was implemented to maintain the simulation system at a constant temperature.^32^ One hundred and ninety‐two peptides were initially randomly placed in a cubic box with a length of 321.0 Å, corresponding to a peptide concentration of 10 mM. The reduced temperature is defined to be *T* ^*^ = *k*_B_ *T*/*ε*_HB_, where *ε*_HB_ = 12.47 kJ/mol is the hydrogen bonding energy. The reduced temperature *T* ^*^ of the simulations was set to be 0.193, which corresponds to 326 K in real temperature units.^33^

## 3 RESULTS AND DISCUSSIONS

### 3.1 Time course of Aβ_16‐22_ aggregation

**FIGURE 1:**
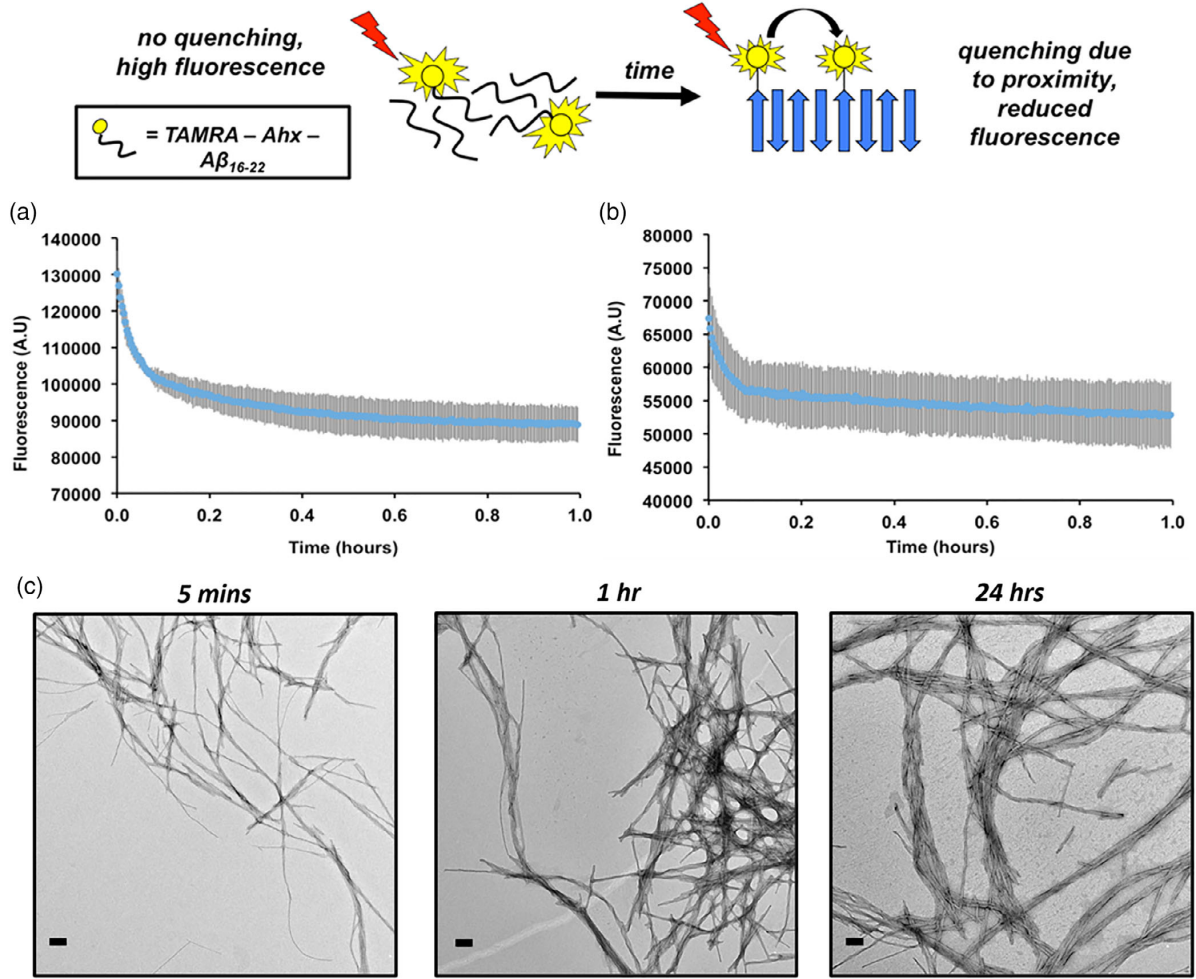
Schematic of the fluorescence quenching assay used in this work. As TAMRA‐Ahx‐Aβ_16‐22_ is incorporated into the fibrils the fluorophores come into close contact and fluorescence is quenched. The aggregation kinetics of Aβ_16‐22_ is monitored using fluorescence quenching. At both 40 and 20 μM (a and b, respectively), Aβ_16‐22_ displays two‐phase aggregation kinetics with the quenching reaching a plateau after around 1 h (100 mM ammonium bicarbonate buffer pH 7.4 with a final concentration of 2% (v/v) DMSO). In a TEM time course (c), Aβ_16‐22_ (40 μM) forms highly ordered fibril structures within 5 min. Scale bar = 200 nm [Color figure can be viewed at wileyonlinelibrary.com]

To monitor the time course for Aβ_16‐22_ aggregation, we used a fluorescence quenching assay (Figure 1) in which sub‐stoichiometric addition of a fluorescently labeled peptide, Aβ_16–22_ N‐terminally labeled with tetramethylrhodamine (TAMRA) including a 6‐aminohexanoic acid linker (Ahx); TAMRA‐Ahx‐Aβ_16‐22_, was included in the Aβ_16‐22_ assembly reaction (Aβ_16–22_/5% (w/w) TAMRA‐Ahx‐Aβ_16–22_ total peptide concentration, 20 or 40 μM). This assay, which is first used to study the aggregation of Aβ_40_ and Aβ_42,_ ^34^ operates on the principle that fluorophores in adjacent peptides in an aggregated state are proximal and therefore self‐quench. We used this assay in our previous study on secondary nucleation of Aβ_40_ by Aβ_16‐22_.^25^ Here, two different concentrations of Aβ_16‐22_ were tested (20 and 40 μM); both self‐assembly reactions (Figure 1a,b) proceed with a rapid initial decrease in fluorescence intensity followed by a second, slower, phase that reaches a plateau after around 1 hr.

Although a powerful method with which to measure the kinetics of peptide self‐association in amyloid formation, fluorescence quenching is limited in that it reports only on the proximity of the fluorophores, but does not provide any information about the underlying structure(s) formed. To further understand the aggregation process of Aβ_16‐22_, samples were taken during the aggregation time course after 5 min, 1 hr, and 24 hr, and analyzed by TEM (Figure 1c). At 5 min fibrils were observed with a small amount of amorphous aggregates also present. After a 1 hr incubation at 37°C, no amorphous aggregates could be seen and increased numbers of fibrils were observed on the grids. At these early time points, the fibrils were well dispersed without significant bundling, whereas at later time points (e.g., after 24 hr) significant bundling of the Aβ_16‐22_ fibrils was observed. The results show that under the conditions employed in this study, Aβ_16‐22_ forms fibril structures rapidly (within ~5 min), with increased numbers of fibrils formed at later times bundling together as the self‐assembly reaction proceeds.

### 3.2 Using ESI‐IMS‐MS and PIC to monitor structures formed during Aβ_16‐22_ aggregation

**FIGURE 2:**
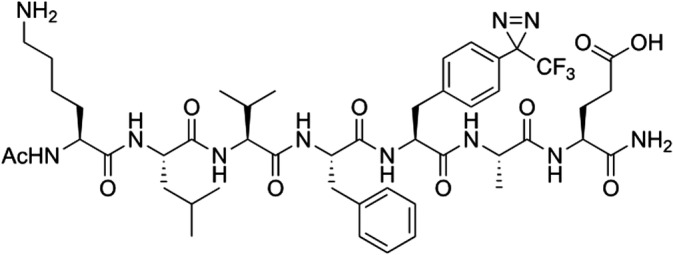
The molecular structure of Aβ*_16–22_

After establishing the aggregation kinetics of Aβ_16‐22_, the next step was to probe the structure(s) that Aβ_16‐22_ accesses during its transition from a monomer to a highly ordered β‐sheet lattice within amyloid‐like fibrils. This was achieved using Aβ_16‐22_ functionalized with 4‐(trifluormethyldiazirine)phenylalanine (TFMD)Phe (named herein as Aβ*_16–22_). The requisite amino acid (TFMD‐Phe) and peptide labeled with (TFMD)Phe at position 20 (Aβ*_16–22_, Figure 2) were prepared as described previously (Section 2).^25^, ^26^ Importantly, we have shown previously that the incorporation of (TFMD)Phe at positions 19 and 20 in Aβ_16‐22_ does not impede aggregation of the peptide nor, in the case of the F20 variant, does it alter the structure of the fibrils formed.^25^, ^26^ We reconfirmed these observations under the conditions used in this work; showing that Aβ*_16–22_ aggregates to yield morphologically similar fibrils to those observed for Aβ_16‐22_ (Figure S1).

### 3.3 Analysis of cross‐linked products

Cross‐linking studies focused on homomeric assembly reactions of Aβ*_16–22_, that is, every peptide in the sample contained a TFMD group at position Phe20. On photolysis, (TFMD)Phe produces a highly reactive carbene which can insert rapidly and indiscriminately into proximal bonds to form a permanent covalent link.^28^, ^35^ As we have shown previously, such cross‐linking approaches can yield information on noncovalent organization in self‐assembled structures.^26^, ^27^ Figures S1b and c show the ion mobility spectrometry coupled to conventional mass spectrometry (IMS‐MS) analyses of the PIC experiment performed on Aβ*_16–22_ after 2 weeks incubation at which time (Figure S1C) fibrils are formed. The resulting mass spectrum identified five major species as photolysis products (Figure S1c). Importantly, the IMS function on the mass spectrometer can provide additional information as it facilitates separation of ions with similar *m*/*z* ratios; cross‐linked monomer, dimer and small amounts of trimer could be observed within the sample, in agreement with our previously published studies (Figure S1B).^26^, ^27^

**FIGURE 3:**
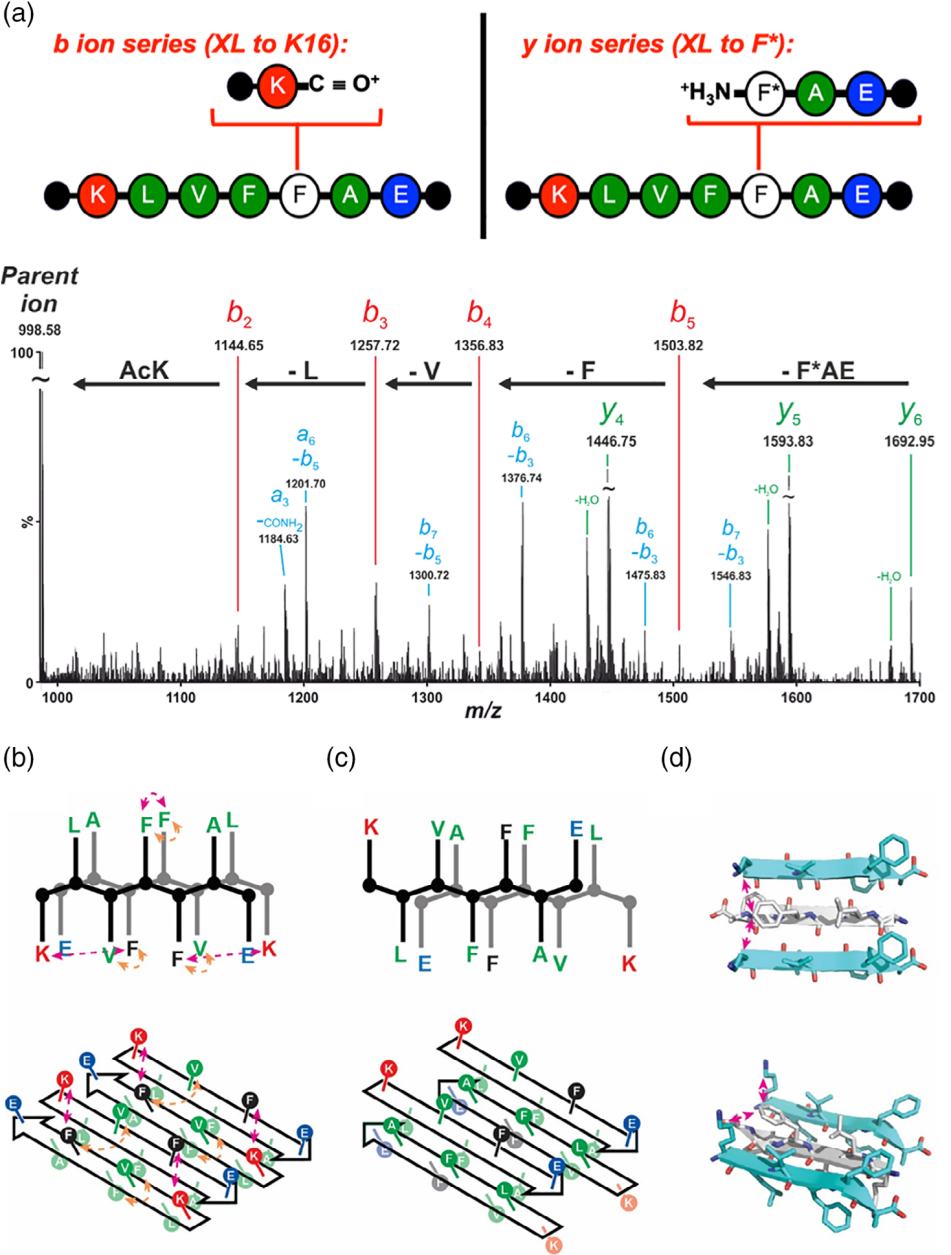
Aβ*_16–22_ forms antiparallel, in‐register β‐sheets after 2 weeks incubation time. (a) The fully assigned tandem MS/MS spectrum for the peak at m/z 988.58 (2^+^; mass 1975.16 Da) contains *b* ions (red, e.g., at 1503.82 corresponding to the loss of F*AE i.e., TFMD‐Phe‐Ala‐Glu), *y* ions (green, e.g., at 1692.95 corresponding to the lost of Lys‐Leu) and double fragmentation products (blue). The two major fragmentation series (*b* and *y* ion) located the cross‐link position to Lys16 and Phe20 on the acceptor chain respectively. Both of these cross‐link patterns are consistent with an antiparallel in‐register β‐strand assembly. Cartoon schematic (b) for in‐register antiparallel and (c) out‐of‐register antiparallel organization of strands (top shows side, bottom shows angle from above, pink arrows illustrate crosslinks observed in this or earlier studies, salmon arrows show contacts observed from MD simulations [see later]). (d) Structure of Lys‐Val‐Leu‐Phe‐Phe‐Ala depicting in‐register antiparallel organization of strands highlighting proximity of Phe and Lys (pink arrows) between adjacent strands (alternate strands shown in cyan and top shows above, bottom shows angle from above) [Color figure can be viewed at wileyonlinelibrary.com]

Tandem MS/MS sequencing fragments a peptide along its amide backbone, producing a variety of different fragment ions.^36^, ^37^, ^38^, ^39^ MS/MS analyses of cross‐linked peptides allow the site(s) of crosslinking to be identified. While the peptide backbone experiences fragmentation, inter‐ and intramolecular crosslinks do not, and so the covalent connectivity between cross‐linked peptide products is preserved and their location can be established from the fragmentation pattern. Consistent with our previous studies,^26^, ^27^ the salient conclusions of this analysis are that PIC of the Aβ*_16–22_ fibrils (Figure 3a) results in formation of intramolecular cross‐links between Phe20 and Glu22, together with intermolecular cross‐links between Phe20 and Lys16. These data are consistent with an assembly whereby noncovalent interactions organize peptide monomers as in‐register anti‐parallel β‐strands in Aβ_16‐22_, fibrils (according to definitions described by Eisenberg and Sawaya^40^), in agreement with our earlier studies.^26^, ^27^ In such an arrangement, Phe20 is directly opposite Val18 on the adjacent strand with Lys16 diagonal to Phe20 (Figure 3b). However, rotation about the α‐β bond allows side chains to adopt a range of orientations, and can be easily visualized in the structure of a related peptide Lys‐Val‐Leu‐Phe‐Phe‐Ala (PDB ID: 2AYA)^41^ whereby the corresponding Phe and Lys side‐chains are in mutual proximity and would be expected to cross‐link more easily. Such a relationship would not be possible for alternate configurations (see Figure 3c for antiparallel in‐register and Supporting Information for further alternatives). Moreover subtle differences in reactivity preference of the carbene arising from diazirine photolysis could also lead to preferential reaction with Lys16 rather than Val18.

### 3.4 Aβ_16‐22_ forms anti‐parallel, in‐register β‐sheets in early protein assemblies

**FIGURE 4:**
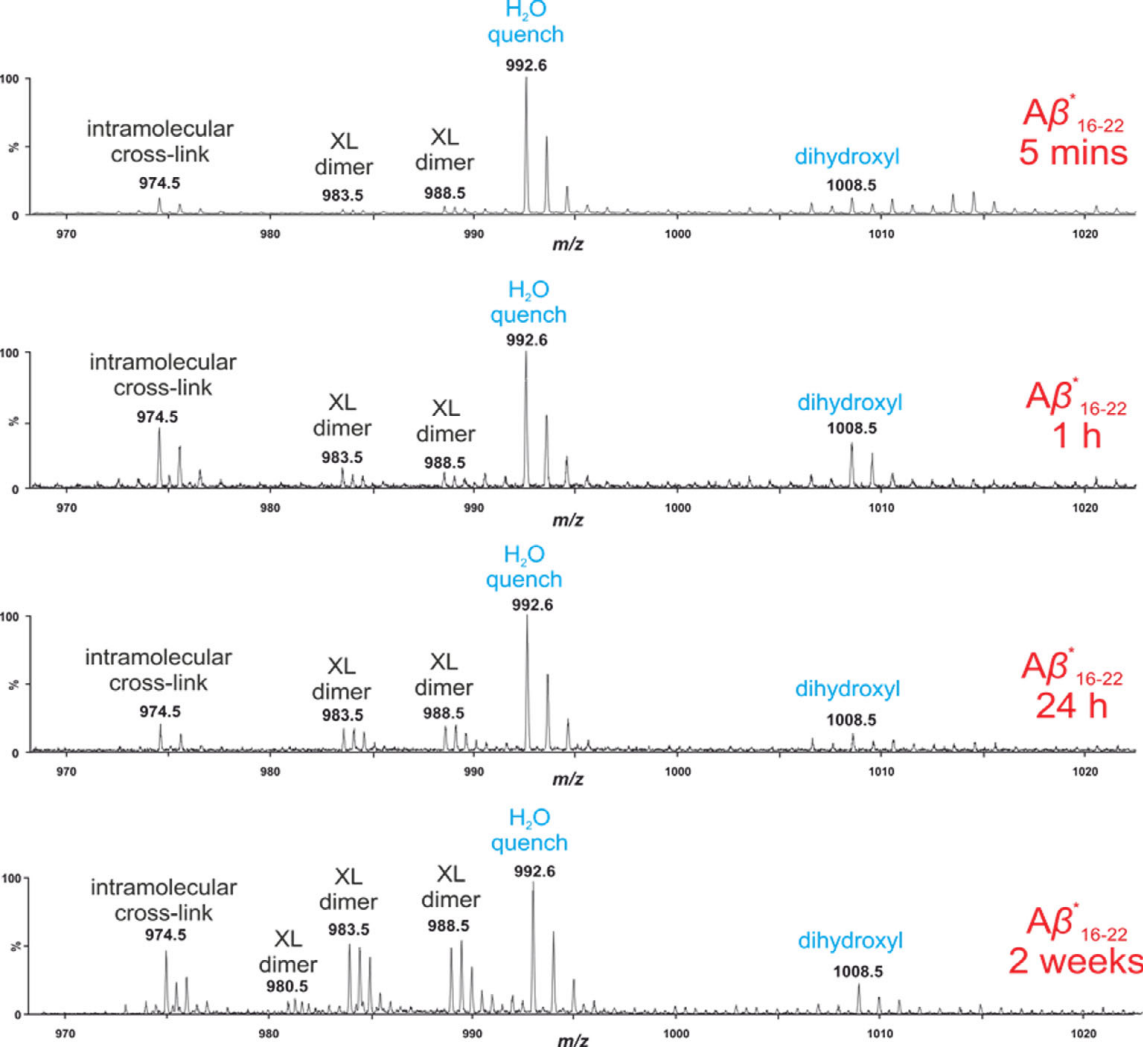
At all time points analyzed Aβ*_16–22_ forms both intra‐ and intermolecular peptide cross‐links. Products from the reaction between the carbene and H_2_O are labeled in blue and peptide cross‐links are labeled in black [Color figure can be viewed at wileyonlinelibrary.com]

**FIGURE 5:**
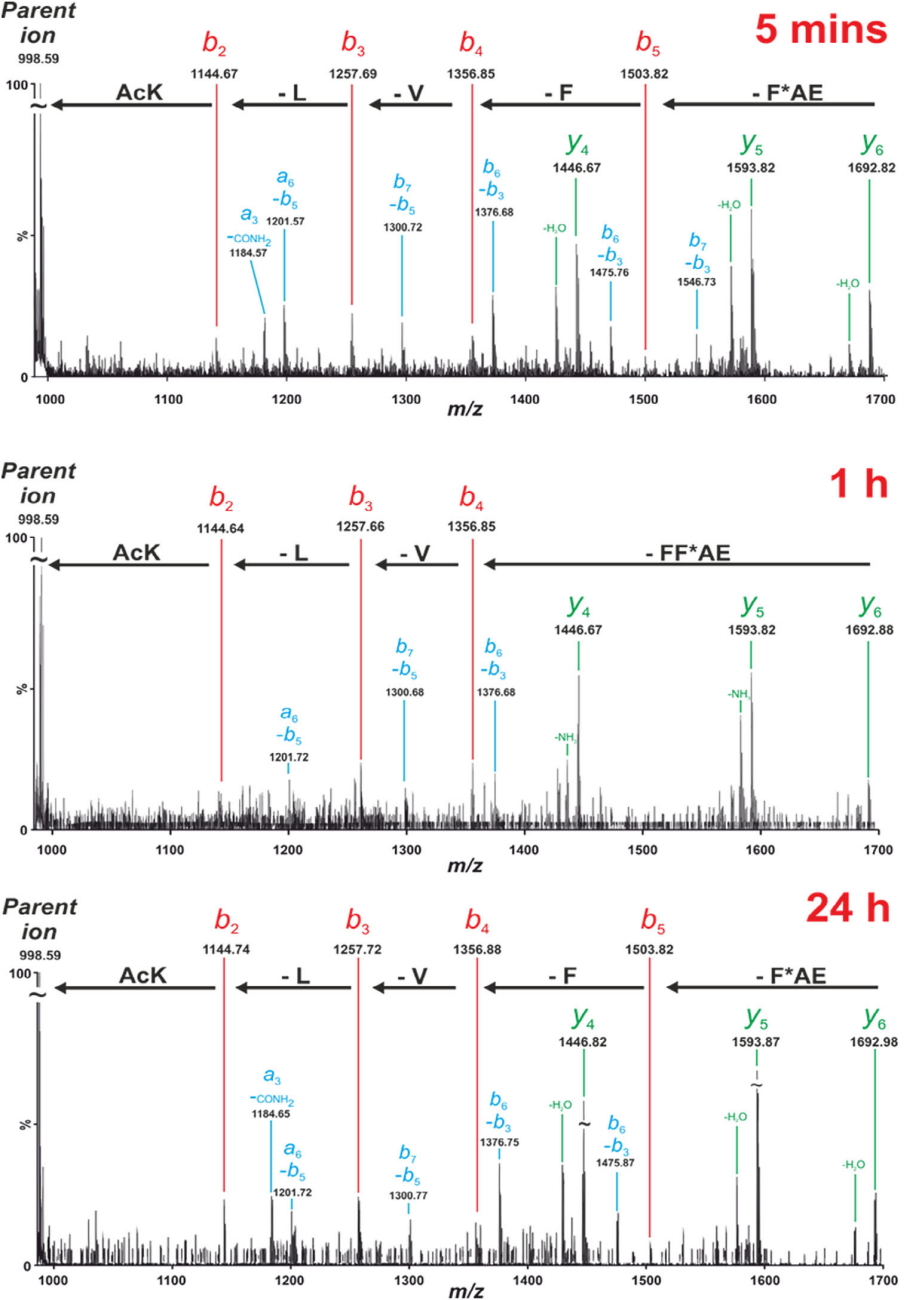
Annotated tandem MS/MS spectra demonstrating that at all time points analyzed Aβ*_16–22_ forms anti‐parallel, in‐register β‐strands. In the spectra, *b* ions are highlighted in red, *y* ions in green and any double fragmentation products in blue [Color figure can be viewed at wileyonlinelibrary.com]

The mass spectrum of cross‐linked products from Aβ*_16–22_ assembly taken at different time points (5 min, 1 hr, 24 hr, and 2 weeks) is shown in Figure 4. Intermolecular cross‐links can be seen at all time points, with the intensity (relative to the H_2_O quenched Aβ_16‐22_) increasing as time progresses. This could be attributed to an increase in the amount of fibrils formed as the incubation time is increased, or the bundling of the Aβ_16‐22_ fibrils after aggregation is complete. Both would increase the concentration of “dry interfaces” from which H_2_O is excluded, allowing the carbene to react with peptide side‐chains, rather than being quenched with H_2_O to form a hydroxyl group.^35^ At each time point, both dominant intermolecular cross‐link peaks (i.e., m/z 998.58 and 983.50) were isolated and sequenced by MS/MS. The results from sequencing the peak at m/z 988.58 (Figure 5) are consistent with those established from sequencing of the disaggregated fibrils after 2 weeks, that is, they indicate the presence of β‐sheet structure in which monomers are organized as in register anti‐parallel β‐strands. The absence of any other cross‐links can be interpreted as follows: (a) Aβ_16‐22_ assembly proceeds through oligomers in which monomers are aligned as anti‐parallel, in‐register β‐strands akin to those in the fibrillar state or (b) cross‐linking reports only on oligomeric and fibrillar assemblies where monomers are assembled as anti‐parallel in register β‐strands, with the remaining species being either too heterogeneous to be sufficiently populated for detection by mass‐spectrometry or too disordered to generate cross‐links other than to water and buffer components.

### 3.5 Large scale DMD simulations of Aβ_16‐22_ assembly

**FIGURE 6:**
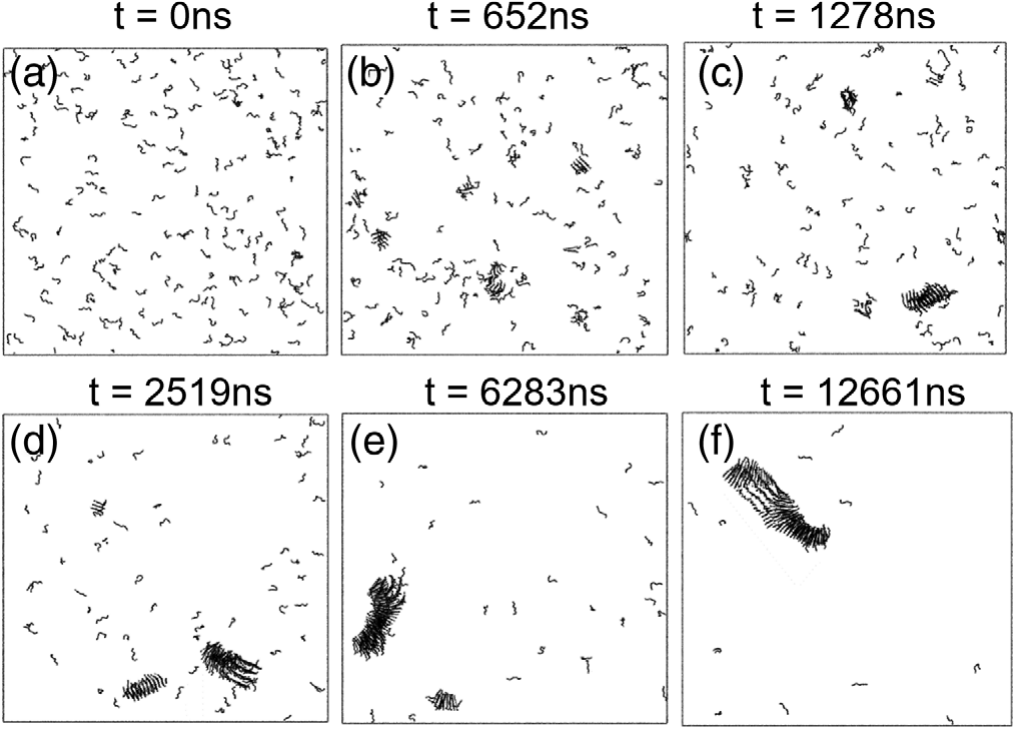
DMD simulation of 192 Aβ_16‐22_ peptides at *T* ^*^ = 0.193 (*T* = 326 K). Snapshots of the system were taken at 0 ns (a), 652 ns (b), 1,278 ns (c), 2,519 ns (d), 6,283 ns (e) and 12,661 ns (f)

To help determine the nature of oligomers formed during Aβ_16‐22_ assembly, DMD and PRIME20 simulations were performed to visualize the transitions that occur during the self‐assembly process. The simulations were conducted as described in Section 2 and a series of simulation snapshots were taken at different time points, as shown in Figure 6. After 652 ns of simulation time, most peptides were still in a random coil conformation with some disordered aggregates and small amounts of ordered oligomers present. As the simulation progressed (1,278 ns), the formation of an anti‐parallel, in‐register oligomer could be clearly seen, as well as a small amount of disordered aggregates. This is in excellent agreement with the structures observed by TEM at early time points (~5 min) where both fibrils and amorphous aggregates are present. At later time points (2,519 and 6,283 ns), most peptides were in ordered aggregates with anti‐parallel, in‐register structures dominating. A single, four‐layered β‐sheet structure was present at the simulation end point (12,661 ns). Again, these results are in close agreement with the experimental data, in which both ordered fibrils and amorphous aggregates were observed at early time points (presumably after a hydrophobic collapse of the peptides upon solvation), prior to the disappearance of the amorphous aggregates and continued formation of fibrils.

**FIGURE 7:**
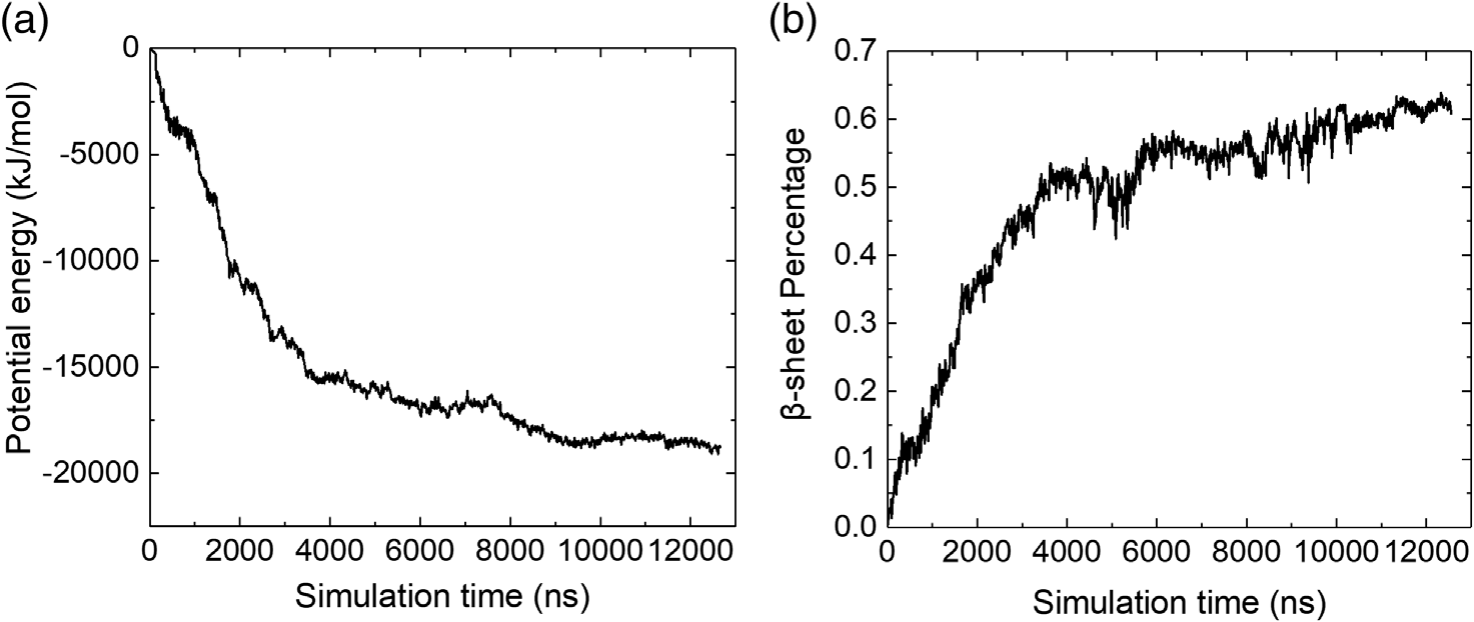
(a) System potential energy profile versus time and (b) Percentage of residues that adopt β‐sheet structures in the system versus time

The kinetics of Aβ_16‐22_ aggregation can be measured in the simulation by calculating the percentage of residues that are in a β‐sheet conformation at different simulation times. Figure 7 demonstrates that, in agreement with the fluorescence quenching data, aggregation proceeds via a rapid increase in β‐sheet content, as measured by the decrease in the system potential energy (from 0 to 50% in the first 4,000 ns) followed by a slower second phase (4,000–12,000 ns). The TEM time course (Figure 1) and the simulation snapshots (Figure 6; i.e., hydrophobic collapse and the presence of small amounts of fibrils at early time points followed by a transition to/continued fibril formation) are concordant, as is the two phase kinetics observed in both the fluorescence quenching and the simulations.

### 3.6 Side‐chain contacts formed during DMD simulations of Aβ_16‐22_ self‐assembly

**FIGURE 8:**
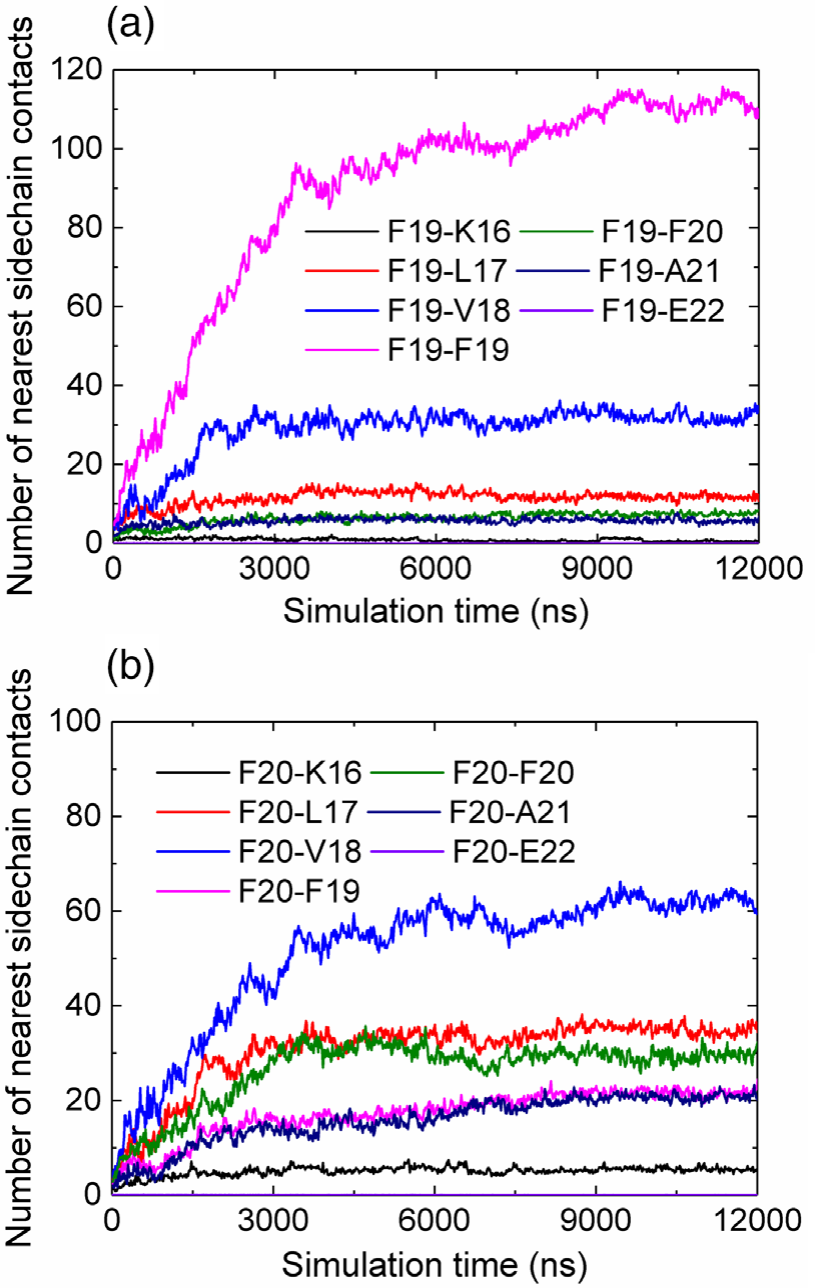
Analysis of the closest interpeptide sidechain contacts formed by F19 (a) and F20 (b) with the other residues of Aβ_16‐22_ in the system as a function of simulation time [Color figure can be viewed at wileyonlinelibrary.com]

The DMD/PRIME20 simulations approach taken in this study can also be used to assess the distance between the beads which represent specific side‐chains beads as the simulation progresses and may be able to identify structures that cannot be resolved in the PIC experiment. To do this, the nearest interpeptide sidechain contacts for all Phe19 and Phe20 residues in the system are calculated and shown in Figure 8. Figure S2 shows a schematic illustrating the simplest anti‐parallel/ parallel in‐register/out‐of‐register potential arrangements that can be envisioned. At the simulation end point, both Phe19 and Phe20 make the most contacts with the residues that are directly opposite their side‐chains in an anti‐parallel, in‐register orientation (Phe19 and Val18, respectively, see Figure 3). Although in isolation the Phe19‐Phe19 contact observed in the simulation would be consistent with both parallel and anti‐parallel orientations of monomers as in‐register β‐strands, in combination with the Phe20 data, the identified contacts are most consistent with an anti‐parallel, in‐register orientation as the dominant mode of interaction. The discrepancy between these MD simulations and the PIC experiments where a cross‐link between Lys16 and Phe20 dominates is reconciled by the fact that PRIME 20 models amino acid side chains as spheres, thus such rotational preferences are not resolved and, instead, a simple distance relationship is observed. Within the data, three distinct phases are observed, which occur on the 0–2,000, 2,000–4,000, and 4,000–12,000 ns timescales. As can be seen in Figure 8a, the second most frequent contact that residue Phe19 makes during the simulation is with Val18 (curve labeled Phe19‐Val18). This contact increased steadily until ~2,000 ns, at which point no further increase occurred. Contact between Phe19 and Val18 would be possible in a parallel out‐of‐register conformation (Figure S2). When analyzing the contacts that Phe20 forms during this initial period (Figure 8b), two significant contacts (other than the dominant Phe20‐Val18 contact) are observed: Phe20‐Leu17 and Phe20‐Phe20. Phe20‐Leu17 interaction would not occur for any of the simplest monomer organizations; this contact again continued to increase in population until ~2,000 ns at which time the number of contacts remained stable for the rest of the simulation. The Phe20‐Phe20 contact, possible in a parallel‐in‐register alignment (Figure S2), increased at a slightly slower rate than the Phe20‐Leu17 contact in the first phase. However, rather than stopping at 2000 ns, it continued to increase until it plateaued at ~4,000 ns. Taken together, the results suggest that as the simulation progresses, an anti‐parallel in‐register β‐sheet forms, while a plethora of alternative structures are also present including disordered aggregates (particularly at early time points), where a heterogeneous distribution of sidechain contacts would be expected. After 2000 ns, however, the anti‐parallel, in‐register alignment starts to dominate, while the contacts for other alignments plateau, indicating that these structures no longer grow, and may interconvert to the in‐register alignment, in agreement with the experimental observations made by Lynn and co‐workers.^13^ These series of simulations demonstrate the power of combining simulations and experimental data to gain a fuller picture of peptide self‐assembly at an atomistic level.

### 3.7 Proposed mechanism of aggregation and comparison with previously proposed Aβ_16‐22_ aggregation mechanisms

**FIGURE 9:**
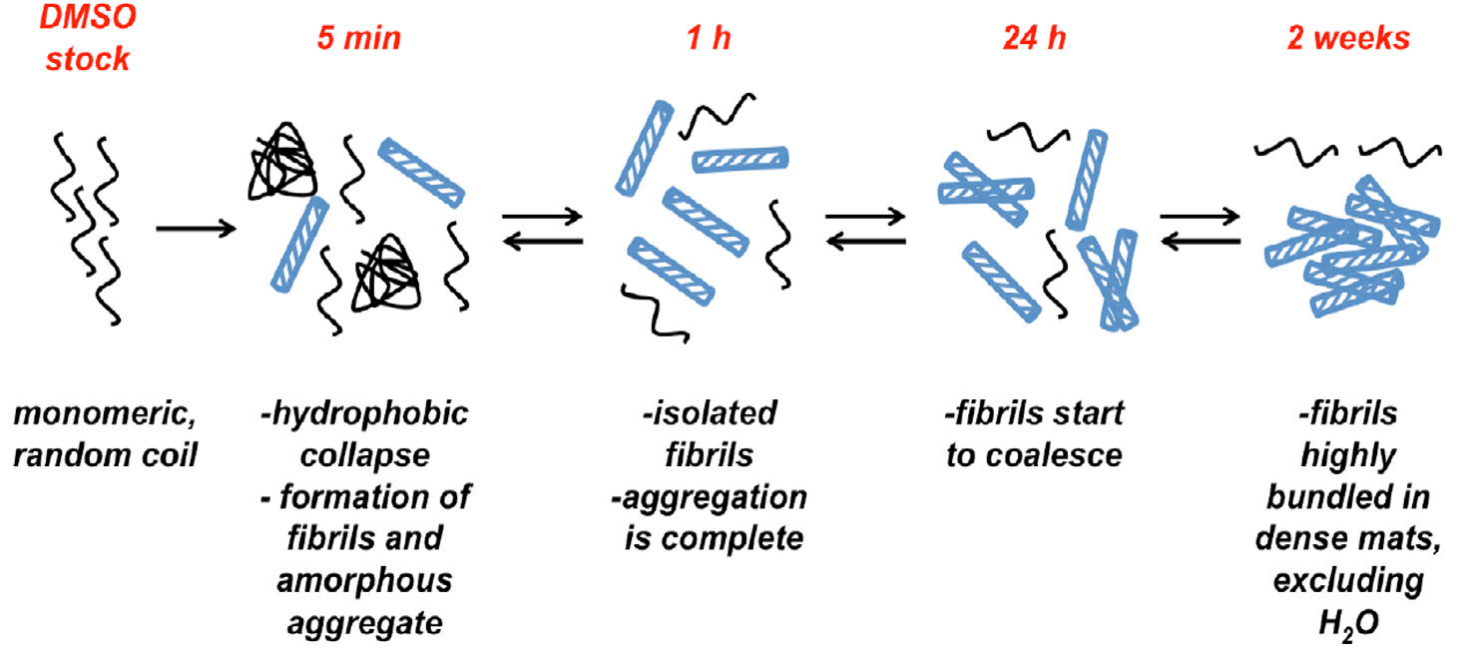
The proposed mechanism for Aβ_16‐22_ self‐assembly [Color figure can be viewed at wileyonlinelibrary.com]

According to the experimental data, the following mechanism for Aβ_16‐22_ self‐assembly is proposed (Figure 9): peptides aggregate rapidly, forming both fibrils and small amounts of amorphous aggregates (the initial decrease in fluorescence, ~ 5 min). These amorphous aggregates then form fibrils, with most of the self‐assembly reaction completed within 1–2 hr (as evidenced by the plateau in the fluorescence quenching data). The fibrils formed at these time points tend to be isolated and unbundled. As the self‐assembly reaction continues, the fibrils start to bundle together and coalesce, forming dense mats of fibril structures (after 2 weeks). These large mats (partly) exclude H_2_O, forming a series of dry interfaces, in turn reducing the opportunity for H_2_O to quench the carbene and promoting the formation of interpeptide cross‐links.

Lynn and co‐workers previously observed a transition from out‐of‐register, anti‐parallel β‐sheets before Aβ_16‐22_ assembles into its final in‐register alignment.^13^ No such transition was discernable in our cross‐linking data. There could be a number of reasons for this:

1. The out‐of‐register alignment may be lowly populated compared with the in‐register alignment and as such may not be captured by the cross‐linking experiments at early time points; this is supported by our simulations.
2. The experimental conditions in this study and the previously reported work of Lynn and co‐workers were different. The prior publication focused on self‐assembly carried out in H_2_O and 40% MeCN with no added salt (i.e., at very low ionic strength), and at higher Aβ_16‐22_ concentration (1.3 mM)^42^ whereas in the present study self‐assembly was carried out in 100 mM ammonium bicarbonate buffer with a peptide concentration of 40 μM. This could cause the out‐of‐register alignment to be disfavored and therefore bypassed, or to be present only transiently prior to the timepoint at which useful cross‐linking data could be obtained (i.e., 5 min).

## 4 CONCLUSION

In this work, Aβ_16‐22,_ a TAMRA‐labeled Aβ_16‐22_ peptide variant and an Aβ*_16–22_ peptide variant were synthesized and used in fluorescence quenching and PIC assays to demonstrate that Aβ_16‐22_ aggregates in two distinct phases. In the first phase, the peptides self‐assemble into β‐sheet assemblies causing the fluorescence intensity to decrease rapidly (~10 min). This is followed by a slower, second phase that plateaus after 1–2 hr. A TEM time course confirmed that fibrils are present after 5 min. To characterize the structures at a number of different time points, the PIC reagent Fmoc‐TFMD‐Phe was synthesized and incorporated into Aβ*_16–22_. PIC with ESI‐IMS‐MS/MS at different time points confirmed that Aβ*_16–22_ forms assemblies in which monomers are organized as anti‐parallel, in‐register β‐strands at all time points. These experimental results were then compared with a series of detailed DMD simulations that are in agreement with the experimental data and allow a molecular mechanism to be proposed for Aβ_16‐22_ assembly under the conditions used in this study, where the dominant pathways involve oligomers where monomers are organized into anti‐parallel, in‐register β‐strands. The DMD results also highlight the presence of intermediary structures that could not be trapped by PIC. Together, the results demonstrate that the combination of crosslinking and MD represents a powerful toolkit with which to visualize the mechanisms of peptide aggregation in molecular and kinetic detail. Such approaches provide proof‐of‐concept for the application of these methods to the study of: (i) disease relevant amyloidogenic peptides (e.g., in Alzheimer's and Parkinson's disease) and (ii) peptide materials.

## AUTHOR CONTRIBUTIONS

**Samuel Bunce:** Conceptualization; data curation; formal analysis; investigation; methodology; resources; validation; visualization; writing‐original draft. **Yiming Wang:** Data curation; formal analysis; investigation; methodology; writing‐original draft; writing‐review and editing. **Sheena Radford:** Funding acquisition; supervision; writing‐review and editing. **Andrew Wilson:** Funding acquisition; project administration; resources; supervision; validation; visualization; writing‐original draft; writing‐review and editing. **Carol Hall:** Funding acquisition; investigation; project administration; supervision; writing‐review and editing.

**NOTATION**
Aβ: amyloid β
DMD: discontinuous molecular dynamics
ESI‐IMS‐MS: electrospray ionization ion‐mobility spectrometry mass‐spectrometry
PIC: photo‐induced crosslinking
TEM: transmission electron microscopy

## Supporting information


**Appendix** **S1**: Supporting Information

## Data Availability

Data in addition to those in the supplementary material are available on request.
