# Supplementary material for: Structural insights into peptide self‐assembly using photo‐induced crosslinking experiments and discontinuous molecular dynamics
Source: AIChE J. 2020 Nov 7;67(3):e17101. doi: 10.1002/aic.17101 (PMC7988534; doi:10.1002/aic.17101)
Supplement: Supplementary file 1 — Appendix S1: Supporting Information [file AIC-67-e17101-s001.docx]

**Supporting information**

**Structural insights into peptide self-assembly using photo-induced crosslinking experiments and discontinuous molecular dynamics**

**Samuel J. Bunce^a,b,†^, Yiming Wang^c,d,†^, Sheena E. Radford^b,e^, Andrew J. Wilson^a,b^, Carol K. Hall^c,*^**

^a^School of Chemistry, University of Leeds, LS2 9JT Leeds, UK

^b^Astbury Centre for Structural Molecular Biology, University of Leeds, LS2 9JT Leeds, UK

^c^Department of Chemical and Biomolecular Engineering, North Carolina State University, Raleigh, NC 27695-7905, USA

^d^Current position: Department of Chemical and Biological Engineering, Princeton University, Princeton, NJ 08544, USA

^e^ School of Molecular and Cellular Biology, University of Leeds, LS2 9JT Leeds, UK

^†^These Authors contributed equally to this work

*Corresponding author: Carol K Hall

Email:  [hall@ncsu.edu](mailto:hall@ncsu.edu)

**Brief Explanation of MS/MS sequencing**

For a more detailed explanation see: ref 1. Tandem MS/MS sequencing fragments isolated peptides along the amide backbone, producing a variety of different fragment ions.[2-5] For larger peptides *b* and *y* ions often dominate the spectra, although for shorter peptides other ions (such as *a* ions) can also be observed. The mass difference (Δ_m_) between individual peaks in the MS/MS spectra corresponds to individual amino acid residues and any modifications that may have occurred. Sequential cleavage produced a series of *b* ions, corresponding to the amino sequence of Aβ*_16-22_. Between the *b6* and *b5* ions, the Δ_m_ = 245.1. As the mass of the isolated TFMD-Phe having extruded molecular N_2_ is = 227.1, the Δm of 18.0 would indicate the addition of H_2_O. As such, the peak m/z 992.58 can be identified as a Aβ*_16-22_ that has reacted with H_2_O, forming a hydroxyl on F20.

In the MS/MS spectrum of the second major monomeric product (m/z 974.52, 1+), no b ions can be observed between *b*5 and *b*8, indicating that an intramolecuar cross-link may have formed between F20 and E22. The covalent link formed between the side chains forms a stable region that is not fragmented in the same way as the peptide backbone.

In considering cross-linked dimers, one peptide chain is considered to be the donor chain (i.e. from where the cross-link originates) and the second peptide chain is considered the acceptor chain (i.e. where the cross-link is going too). The overall m/z of the cross-linked dimer identifies the fate of the diazirine on the acceptor chain (e.g. m/z 988.58 indicates that the diazirine has undergone conversion to the linear diazoisomer). Any singly charged peaks above the m/z of the parent ion could come from fragmentation of each chain (i.e. there are multiple isomeric products for certain m/z ratios), however, certain diagnostic peaks can be used to identify the underlying β-sheet alignment. In the system studied here, every Aβ_16-22_ peptide contains TFMD-Phe at position 20. As such, to unambiguously assign the cross-link position to the acceptor chain, the fragment lost must contain the TFMD-Phe residue (i.e. it acts as a diagnostic residue for structural assignment). The possibility of double fragmentation (i.e. fragments are lost from both acceptor and donor chains) can complicate the assignment process. Here we employed identical methods to those described previously. Briefly, once the identity of the residue in the acceptor chain has been elucidated, this information must be compared with the different possible models (i.e. parallel/antiparallel and in/out-of-register) to identify the underlying β-sheet structure. It should be noted that due to the distance constraint imposed by placing the diazirine in the amino acid sequence, only inter-sheet (vs intra-strand) cross-links are possible.

###



Backbone fragmentation nomenclature for the **Aβ_16-22_** monomer and its derivatives. R_1_, R_2_, R_3_, R_4_, R_5_, R_6_ and R_7_ are the side chains of Lys-16, Leu-17, Val-18, Phe-19, Phe-20, Ala-21 and Glu-22 (or derivatives thereof), respectively. Nomenclature is based on the Roepstorff-Fohlman-Biemann convention [3,4], but note that the N-terminal acetyl and C-terminal NH­_2_ moieties are treated as residues for the purposes of assigning backbone fragmentations.

###



Backbone fragment nomenclature for a singly cross-linked dimer, using a **Aβ_16-22_-F20*** derivative as an example. R_1_, R_2_, R_3_, R_4_, R_6_ and R_7_ are the side chains of Lys-16, Leu-17, Val-18, Phe-19, Ala-21 and Glu-22 (or derivatives thereof), respectively. Schilling et al. [5] differentiate the cross-linked peptides as α and β on the basis of mass. Since our cross-linked peptides are always similar in mass, we use a modified nomenclature in which α corresponds to the peptide from which the cross-link has formed and β corresponds to the peptide to which the cross-link has formed. The side group at position R_5_ is variable depending on the fate at the cross-linker at this position. Alternatively, if β is unmodified **Aβ_16-22_**, then R_5_ = H.

**
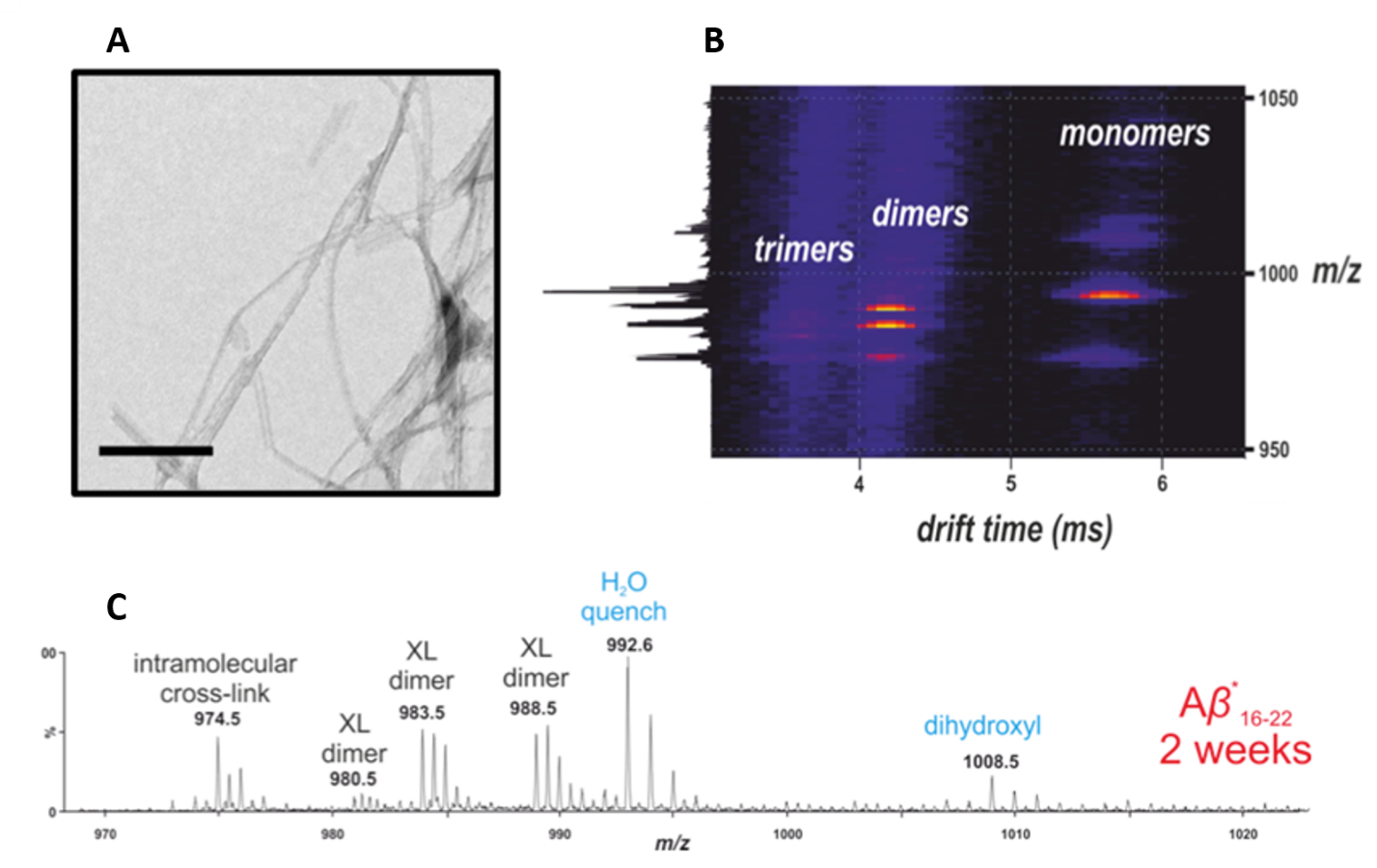
**

**Figure S1**. Control PIC experiment for Aβ*_16-22_, under the conditions used in this study. After 2 weeks incubation, Aβ*_16-22_ forms fibrils that are morphologically similar to those formed by Aβ_16-22_ (A). After irradiation at 365 nm and disaggregation in HFIP, the drift-scope image (B) demonstrates that IMS can resolve crosslinked monomers, dimers and trimers prior to MS/MS analysis. In the mass spectrum, 5 major peaks can be observed (C) (XL denotes cross-linked product, intramolecular cross-link denotes the monomeric product resulting from intramolecular insertion of the carbene (resulting from diazirene photolysis) within the peptide chain, H_2_O quench denotes the monomeric product resulting from insertion of the carbene into water and dihydroxyl denotes the monomeric product resulting from reaction of the carbene to form a ketone which becomes hydrated as described previously). [1]


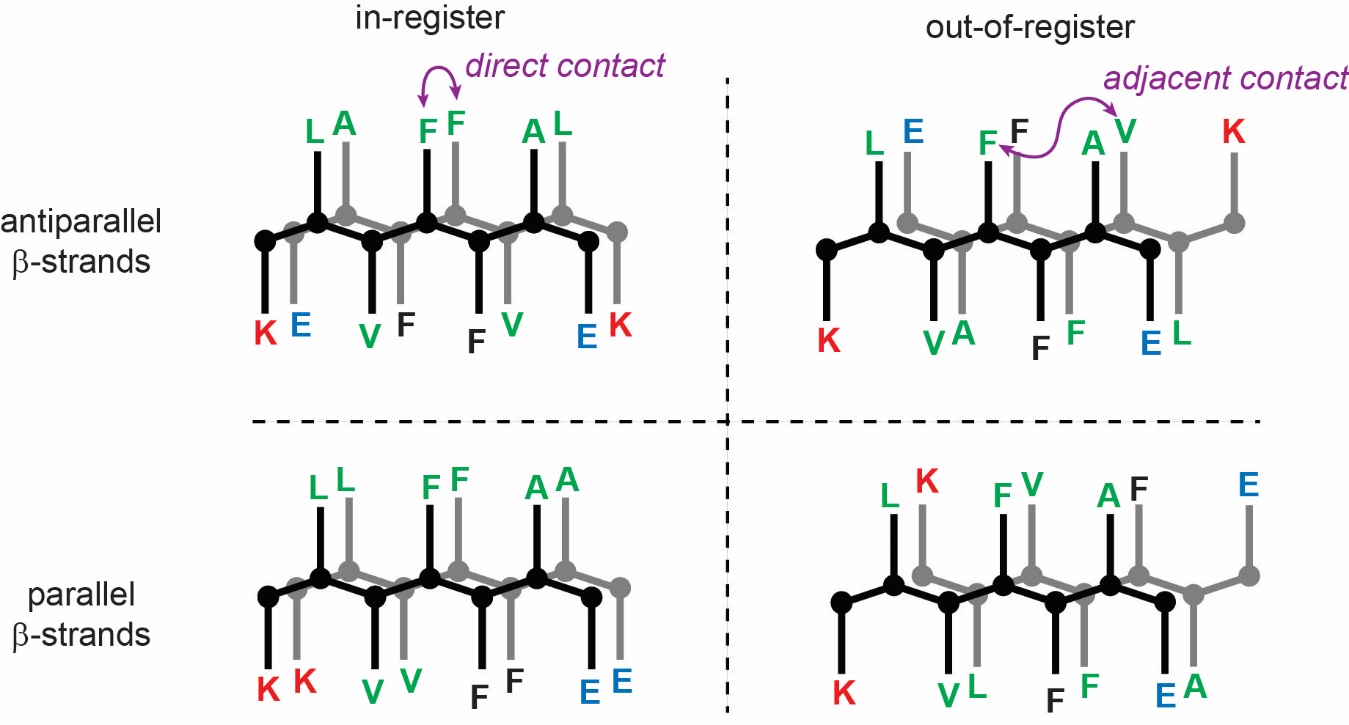


Figure S2. Schematic illustrating different arrangement of side chains for four β-sheet arrangements that Aβ_16-22_ might possibly adopt – direct and adjacent contacts are highlighted in purple, side chains are color coded: green for hydrophobic, red for basic and blue for acidic except for F20 in black


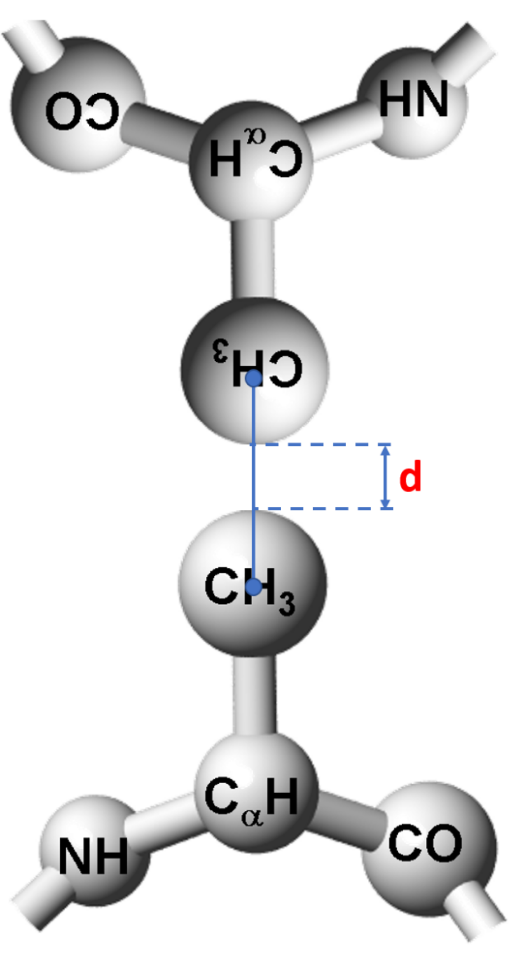


Figure S3. The nearest sidechain contact is determined when a target sidechain bead has the minimum d value with the surrounding neighbor sidechain bead.

**References**

1. Preston GW, Radford SE, Ashcroft AE, Wilson AJ. Covalent cross-linking within supramolecular peptide structures. Anal. Chem. 2012;84:6790–6797.

2. Smith DP, Knapman TW, Campuzano I, Malham RW, Berryman JT, Radford SE, Ashcroft AE. Deciphering drift time measurements from travelling wave ion mobility spectrometry-mass spectrometry studies. Eur. J. Mass Spectrom. 2009;15:113-130.

3. Roepstorff P, Fohlman J. Proposal for a common nomenclature for sequence ions in mass spectra of peptides. Biomed. Mass Spectrom. 1984;11:601-601.

4. Biemann K. Mass spectrometry of peptides and proteins Annu. Rev. Biochem. 1992;61:977-1010.

5. Schilling B, Row RH, Gibson BW, Guo X, Young MM. MS2Assign, automated assignment and nomenclature of tandem mass spectra of chemically crosslinked peptides. J. Am. Soc. Mass Spectrom. 2003;14:834-850.
